# A Potential Role of Flag Leaf Potassium in Conferring Tolerance to Drought-Induced Leaf Senescence in Barley

**DOI:** 10.3389/fpls.2016.00206

**Published:** 2016-02-26

**Authors:** Seyed A. Hosseini, Mohammad R. Hajirezaei, Christiane Seiler, Nese Sreenivasulu, Nicolaus von Wirén

**Affiliations:** ^1^Molecular Plant Nutrition Group, Physiology and Cell Biology, Leibniz-Institute of Plant Genetics and Crop Plant ResearchGatersleben, Germany; ^2^Abiotic Stress Genomics Group, Molecular Genetics, Leibniz-Institute of Plant Genetics and Crop Plant ResearchGatersleben, Germany

**Keywords:** ABA homeostasis, potassium efficiency, drought stress tolerance, primary metabolites, starch metabolism

## Abstract

Terminal drought stress decreases crop yields by inducing abscisic acid (ABA) and premature leaf senescence. As potassium (K) is known to interfere with ABA homeostasis we addressed the question whether there is genetic variability regarding the role of K nutrition in ABA homeostasis and drought tolerance. To compare their response to drought stress, two barley lines contrasting in drought-induced leaf senescence were grown in a pot experiment under high and low K supply for the analysis of flag leaves from the same developmental stage. Relative to the drought-sensitive line LPR, the line HPR retained more K in its flag leaves under low K supply and showed delayed flag leaf senescence under terminal drought stress. High K retention was further associated with a higher leaf water status, a higher concentration of starch and other primary carbon metabolites. With regard to ABA homeostasis, HPR accumulated less ABA but higher levels of the ABA degradation products phaseic acid (PA) and dehydro-PA. Under K deficiency this went along with higher transcript levels of *ABA8*′*-HYDROXYLASE*, encoding a key enzyme in ABA degradation. The present study provides evidence for a positive impact of the K nutritional status on ABA homeostasis and carbohydrate metabolism under drought stress. We conclude that genotypes with a high K nutritional status in the flag leaf show superior drought tolerance by promoting ABA degradation but attenuating starch degradation which delays flag leaf senescence. Flag leaf K levels may thus represent a useful trait for the selection of drought-tolerant barley cultivars.

## Introduction

Drought is one of the most serious environmental stresses limiting crop growth and productivity worldwide (Huang et al., 2014). The severity of drought stress not only depends on the duration and intensity (Samarah et al., 2009) but also on the developmental phase of the plants in which drought stress sets in. Plants may experience drought as transient or terminal stress, which leads to different physiological and developmental responses (Munné-Bosch and Alegre, 2004). If drought stress occurs during the vegetative growth phase, the stress is mostly transient and holds on until rainfall comes back and restores plant growth. This is typical for pre-summer drought periods, which are seen more and more in the continental climates of middle Europe. In this case, plants slow down growth and may even start wilting, while yield formation is mainly limited by suppressed tillering, i.e., a lower number of ear-bearing tillers per plant (Sreenivasulu et al., 2007). Occurrence of drought during generative plant development, i.e., around flowering, may induce premature leaf senescence that leads to a decline in photosynthesis and assimilate allocation, causing acceleration of the whole-plant maturation process (Gan, 2003). Under such terminal drought crop yields are mostly restricted by plant reproductive failure, followed by a shortening of the grain filling period which ultimately reduces grain number and grain size (Sreenivasulu et al., 2007).

An early consequence of low water availability under drought is a decrease in total nutrient uptake and translocation to shoots. A lower absorption of inorganic nutrients results mainly from a reduced transpirational flow and hence a decreased mass flow to the root surface of soil water containing soluble nutrients. Transient periods of drought may therefore decrease the uptake of nitrate and provoke nitrogen (N) deficiency (Marschner, 2012). However, when persisting water scarcity leads to a disconnection of the water film between the roots and the soil matrix, diffusion-driven nutrient transport will cease, which has a large impact on the accessibility to potassium (K) (Kant and Kafkafi, 2002). In an attempt to separate the influences of soil moisture and root growth on K uptake under drought, total K uptake was found to decrease not only as a consequence of the lower root length density but also of the lower rate of K diffusion in dry soil (Barber et al., 1988). A combined application of N, phosphorous (P) and K (NPK) as foliar spray enhanced grain yield in wheat under drought, although it remained open to what extent K nutrition alone improved plant growth (Shabbir et al., 2015). At a larger scale, modeling studies projected the importance of K on gross primary productivity (GPP) of stem biomass in Eucalyptus, where K deficiency under water-limiting conditions reduced GPP by 28% (Christina et al., 2015).

Potassium is an essential mineral element required for plant growth and stress tolerance (Restrepo-Diaz et al., 2008; Wei et al., 2013). In particular the role of K in the maintenance of cell turgor and stomatal regulation is of major importance for drought tolerance (Gupta et al., 1989; Cakmak, 2005). Another important relationship between the K nutritional status and drought stress responses has been demonstrated at the metabolic level, where compatible solutes such as sugars, sugar alcohols and proline warrant osmotic adjustment, permitting the maintenance of cell turgor under conditions of water deficit (Seki et al., 2007). Here, K^+^ activates several enzymes in carbohydrate metabolism incl. pyruvate kinase, phosphofructokinase or starch synthase, which maintain carbohydrate fluxes in photosynthesis and primary metabolism (Marschner, 2012). Using molecular approaches in *Arabidopsis* a link has been established between the signal transduction pathways induced by K deficiency and by drought stress. Potassium deprivation activates a type III-peroxidase responsible for ROS production, which triggers via cytosolic Ca signaling the induction of K transporters in roots as well as in leaves (Cheong et al., 2007; Kim et al., 2010). In roots, this promotes an increase in K uptake capacity and in leaves a more tight regulation of stomatal aperture, for instance by inducing the K^+^ transporter gene *HAK5* and the K^+^ channel gene *AKT1* (Ashley et al., 2006; Kim et al., 2009). Likewise, drought stress employs the same signal transduction chain entering ROS production and Ca signaling most likely via enhanced abscisic acid (ABA) biosynthesis, also leading to the induction of K transporters and channels in roots and guard cells (Cheong et al., 2007). Enhanced levels of ABA have also been reported for K-deficient flag leaves of wheat, supporting the general importance of a cross-talk between ABA and K deficiency signaling (Haeder and Beringer, 1981). By this way, drought stress additionally stimulates the induction of K deficiency responses to maintain transpiration and carbon assimilation. So far, this relation has mainly been addressed at the regulatory level, leaving open which role ABA takes in K deficiency signaling and to what extent the K nutritional status protects carbohydrate metabolism from drought-induced stress in crops.

Crop species and genotypes may differ in their efficiency to take up and use K (Rengel and Damon, 2008). A considerable genotypic variability in K efficiency among genotypes has been reported for barley (Pettersson and Jensén, 1983), canola (Damon et al., 2007), wheat (Damon and Rengel, 2007), and cotton (Chen et al., 2007). In these studies genotypical differences turned out to be mainly related to K utilization efficiency, while only in a few cases K uptake apparently contributed more to K efficiency. Imposing osmotic stress by polyethylene glycol on two hydroponically grown wheat lines revealed that the drought-tolerant genotype performed better than the drought-sensitive genotype regarding leaf water potential or the activity of antioxidant enzymes (Wei et al., 2013). When K supply was increased under drought, both wheat lines showed improved stress tolerance under elevated K supply, but there was no indication for a differential responsiveness of these wheat lines to supplemented K (Wei et al., 2013). Despite the importance of K nutrition in drought tolerance, a direct comparison of drought tolerance in K-efficient and K-inefficient crop cultivars has not yet been reported. Thus, it has remained open, whether K-efficient genotypes may counteract drought stress more effectively and whether this is related to genotypical differences in ABA homeostasis.

To address the role of K in terminal drought stress in barley, we selected two elite barley lines, which differed in their senescence behavior under terminal drought. According to Illumina 9K SNP analysis these so far unpublished lines show approximately 65% genetic identity. These lines were submitted to drought under low or high K supply to investigate the relation between K nutrition and stress metabolism in flag leaves of the same developmental stage. Investigating senescence markers as well as K and metabolite concentrations pointed to a critical role of K in maintaining starch as an important carbon reserve to encounter drought-induced leaf senescence. A closer look at ABA homeostasis in these two contrasting lines showed that the higher K retention in the drought-tolerant line coincided with a higher turnover of ABA. Thus, this study emphasizes a critical role of the flag leaf K nutritional status in preventing drought-induced leaf senescence.

## Materials and Methods

### Plant Material and Growth Conditions

The barley lines (*Hordeum vulgare*) cvs. HPR (high potassium retainer) and LPR (low potassium retainer) are representative inbred lines that were derived from a double haploid breeding program of KWS-Lochow GmbH, Germany. Plants were cultivated in a greenhouse in a 16/8 h light/dark cycle at 20/15°C. A commercial growth substrate (Substrat 1, Klasmann Deilmann GmbH, Germany) was analyzed for mineral element contents (Eurofin, Germany), and the required amounts of nutrients were calculated that were needed to be added to the substrate to sustain plant growth until senescence. Thereafter, 4 weeks-old plants were transferred into 5 l pots filled with 2 kg of peat-based growth substrate fertilized with K. Potassium was supplied at two levels to the substrate: low (no added K) or high (4 g kg^-1^ K_2_SO_4_). In addition to that, the substrate was supplemented with 9 g kg^-1^ CaCO_3_ and 4.05 g kg^-1^ CaO in order to increase the pH of the acidic peat-based substrate. When spikes emerged, all spikes were tagged manually with the date of the beginning of flowering, so that only spikes of the same developmental stage were used for the collection of flag leaves. Drought stress was imposed 5 days after flowering and these plants were maintained at 10% soil moisture content. Twelve days after imposing drought (corresponding to 17 days after flowering) flag leaves were harvested from stress and control plants. Throughout the drought treatment, soil moisture levels were monitored using the moisture meter HH2 with a soil moisture sensor SM200 (Delta T devices). The remaining batch of plants was continuously watered and treated as control.

### Chlorophyll and Shoot K Measurements

For chlorophyll determination 20 mg of fresh flag leaf material was incubated at 4°C for 24 h in N,N9-dimethylformamide (Merck). The absorbance in the extracts was determined at 647 and 664 nm following the protocol described by Porra et al. (1989). For K measurements, flag leaf samples were dried for 48 h at 65°C and digested with HNO_3_ in polytetrafluoroethylene vials in a pressurized microwave digestion system (UltraCLAVE IV, MLS GmbH). Potassium concentrations were analyzed by inductively coupled plasma (ICP)-optical emission spectrometry (iCAP 6500 DualOES Spectrometer, Thermo Fischer Scientific).

### RNA Isolation, cDNA Synthesis, and Gene Expression Analysis

Total RNA was extracted from 100 mg leaf material using TRIzol reagent (Invitrogen, USA), and RNA was purified using the RNayes MinElute cleanup kit (Qiagen, USA) following the manufacturer’s protocol. The quality of RNA was checked on a 1% agarose gel, and RNA concentrations were measured using a photometer (BIO-TEC, UVLKONxl, Germany). Genomic DNA was digested using RQ1 RNase-Free DNase (Promega). 2 μg of total RNA were taken for cDNA synthesis using RevertAid First Strand cDNA Synthesis Kit (Fermentas, St. Leon-Rot, Germany) and oligo-dT-primers. To test cDNA yield, qPCR was performed using primers of the reference gene ubiquitin-conjugating enzyme E2-17 kDa that was stably expressed under the experimental conditions tested. The cDNA samples were used to investigate gene expression by quantitative real-time PCR using a Master cycler ep realplex (Eppendorf) and the iQ SYBR Green Supermix (Bio-Rad Laboratories) and the primers listed in Supplementary Table S1. Relative expression levels were calculated according to Seiler et al. (2011).

### Determination of Primary Metabolites

Soluble sugars and starch were determined in flag leaves according to the method of Chen et al. (2005). 50 mg frozen flag leaf material were homogenized in liquid nitrogen, dissolved in 0.75 ml of 80% (v/v) ethanol and incubated at 80°C for 60 min. Crude extracts were centrifuged at 14,000 rpm and pellets were re-suspended in 0.25 ml HPLC-grade water and shaken for 15 min at 4°C. Hexokinase (HK), phophoglucoisomerase (PGI), and β-fructosidase were added successively to measure Glc, Fru, and Suc as described in Hajirezaei et al. (2000). Starch was decomposed with 0.4 ml of 0.2 N KOH for 16 h at 4°C and neutralized with 70 μl of 1 M acetic acid. Hydrolysis of starch was performed using a 1:1 ratio of sample and a buffer containing 50 mM sodium acetate at pH 5.2, and 7 units mg^-1^ amyloglucosidase (Roche, Germany). The cocktail was incubated at 37°C for 16 h. Determination of produced Glc was performed according to Hajirezaei et al. (2000).

To measure the concentration of sugar alcohols a BioLC DX600 system (Dionex, Idstein, Germany) was used. Separation of the anionic compounds was carried out using a CarboPack MA1 column (4 mm × 250 mm). A linear gradient was accomplished with purest water (buffer A, Millipore) and 480 mM sodium hydroxide (Baker, 50% solution, buffer B). The column was equilibrated at a flow rate of 0.4 ml min^-1^. The calibration and quantitative calculation of metabolites was carried out using the Chromeleon software 6.6.

Free amino acids were extracted as described by Chen et al. (2005) and determined according to Hajirezaei et al. (2000). Quantification of metabolites was performed by creating a batch for each sample set using the Quantitative Analysis (QQQ) software (Agilent Germany). Flag leaves of barley plants were ground in liquid nitrogen and 100 mg of finely powdered fresh material were extracted using 1 ml (1:1, v/v) ice-cold methanol and chloroform. The samples were centrifuged for 10 min at 14000 rpm and 4°C. Thereafter, the upper phase containing methanol/water was transferred to new Eppendorf tubes and concentrated at 45°C for 2 h in a speed vacuum concentrator (Christ ALPHA 2-4 LD plus, Germany). The remaining pellet was used for separation and quantification of primary metabolites by a Dionex ICS5000 (Dionex, Idstein, Germany).

### Phytohormone Measurements

The concentrations of ABA and its degradation products PA and DPA were separated in a UPLC system using a high capacity column (Eclipse Plus C18, RRHD 1.8 μm, 2.1 mm × 50 mm). A gradient was accomplished with LCMS-grade water (Chem solute, Th. Geyer, Germany) containing 0.1% formic acid (Fluka, Germany) as buffer A and LCMS-grade methanol (Chem solute, Th. Geyer, Germany) including 0.1% formic acid as buffer B. The column was equilibrated with a mixture of buffer A (86.5%) and buffer B (13.5%) at a flow rate of 0.4 ml per minute and maintained at 40°C. The gradient was produced by changes of the buffer B as follows: 0–5 min at 18%, 5–6 min at 70%, 6–7 min at 99%, 7–7.1 min at 13.5%, and kept up to 9 min at 13.5%. The whole duration of the run was 9.0 min. Mass spectrometry was performed using a 6490 triple Quad MS-MS (Agilent, Germany). The following parameters were employed: desolvation temperature 350°C, desolvation N_2_ gas of 720 l h^-1^, capillary voltage 2.0 KV, detection in positive ion mode and different dwell times between 40 and 200 s. Protonated ions [M-H]^+^ were monitored with a span of 1 amu. Multiple reactions monitoring (MRM) was performed to identify individual compounds accurately. All experiments were repeated at least once and representative data are shown.

## Results

### HPR and LPR Differ in Drought-Induced Leaf Senescence under Limited K Supply

**FIGURE 1:**
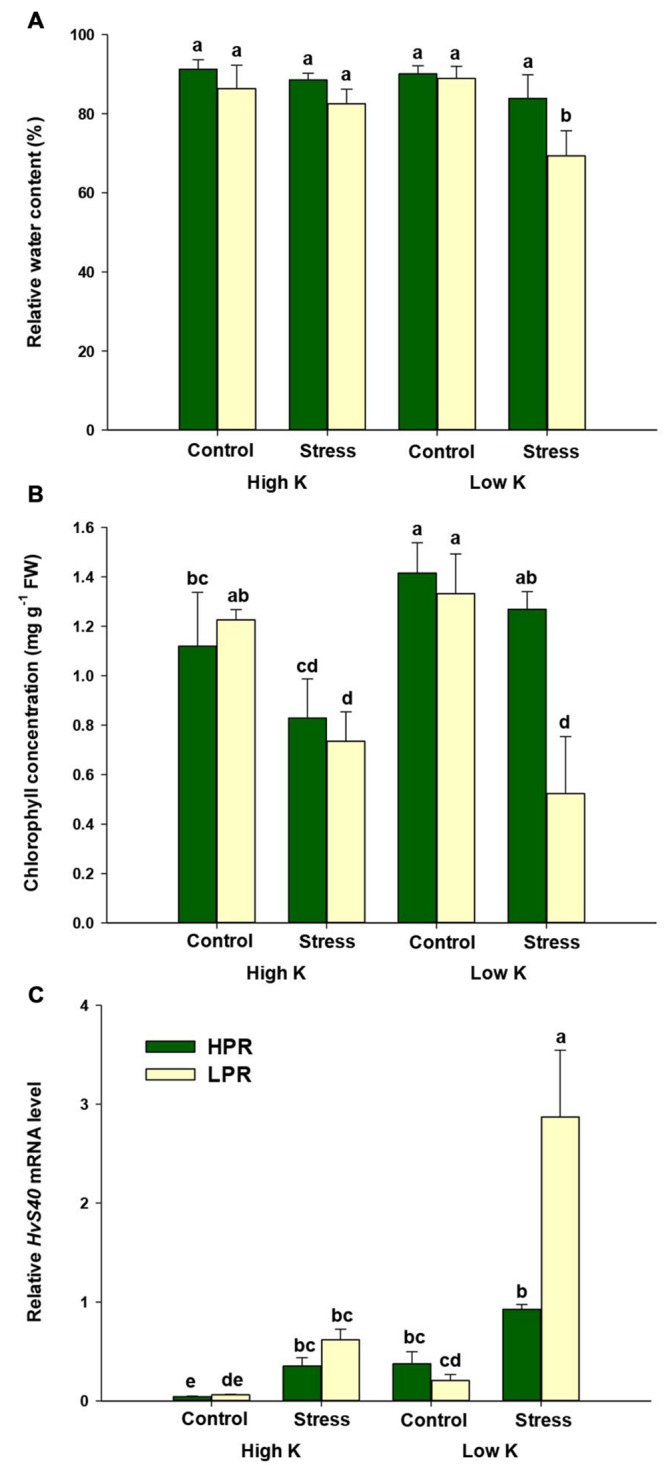
**Influence of K supply on relative water content and senescence parameters in flag leaves of barley during terminal drought stress. (A)** Relative water contents (RWCs), **(B)** chlorophyll concentrations, and **(C)** relative *HvS40* mRNA levels in flag leaves of the lines HPR and LPR. Plants were pre-cultured under sufficient water supply (control) or water limitation (stress) and under low or high K supply. Flag leaves from 12 weeks-old plants were harvested 12 days after imposing drought stress. Bars indicate mean ± SD. Different letters denote significant differences according to three-way ANOVA and Tukey’s test (*p* < 0.05; *n* = 6). *Ubi E2-17* (ubiquitin-conjugating enzyme) was used as reference gene.

In order to determine whether drought-induced leaf senescence is modulated by the K nutritional status, we cultivated two barley lines, which differ in senescence behavior under drought stress, at two K regimes and exposed them to terminal drought stress at flowering. To ensure comparability between the lines, only flag leaves from tillers were analyzed that were in the same developmental stage. Twelve days after imposing drought, the relative water content of flag leaves showed no decrease when plants were grown at adequate K supply (**Figure 1A**). Under low K supply, however, relative water content in flag leaves of the line LPR significantly decreased from 89 to 69%, whereas the corresponding decrease in HPR remained insignificant (90–83%). Thus, flag leaves of HPR showed a higher tolerance to drought-induced water loss under low K supply.

Under ample K supply, chlorophyll concentrations in flag leaves tended to decrease under drought stress in both lines. Under K deficiency, chlorophyll levels dropped severely in the line LPR, while HPR plants maintained their chlorophyll levels despite drought (**Figure 1B**). To confirm whether this drop in chlorophyll levels is related to senescence, we determined by quantitative RT-PCR expression levels of the senescence marker gene *HvS40* (Krupinska et al., 2002). Transcript levels of *HvS40* showed only weak induction under drought stress in K-supplied plants, while there was a much stronger induction by drought under K deficiency but only in LPR (**Figure 1C**). By contrast, in HPR *HvS40* mRNA levels responded poorly to drought despite K deficiency. Thus, both markers chlorophyll and *HvS40* mRNA levels, indicated that the line HPR suffered less from drought-induced leaf senescence than LPR when K supply was limited.

**FIGURE 2:**
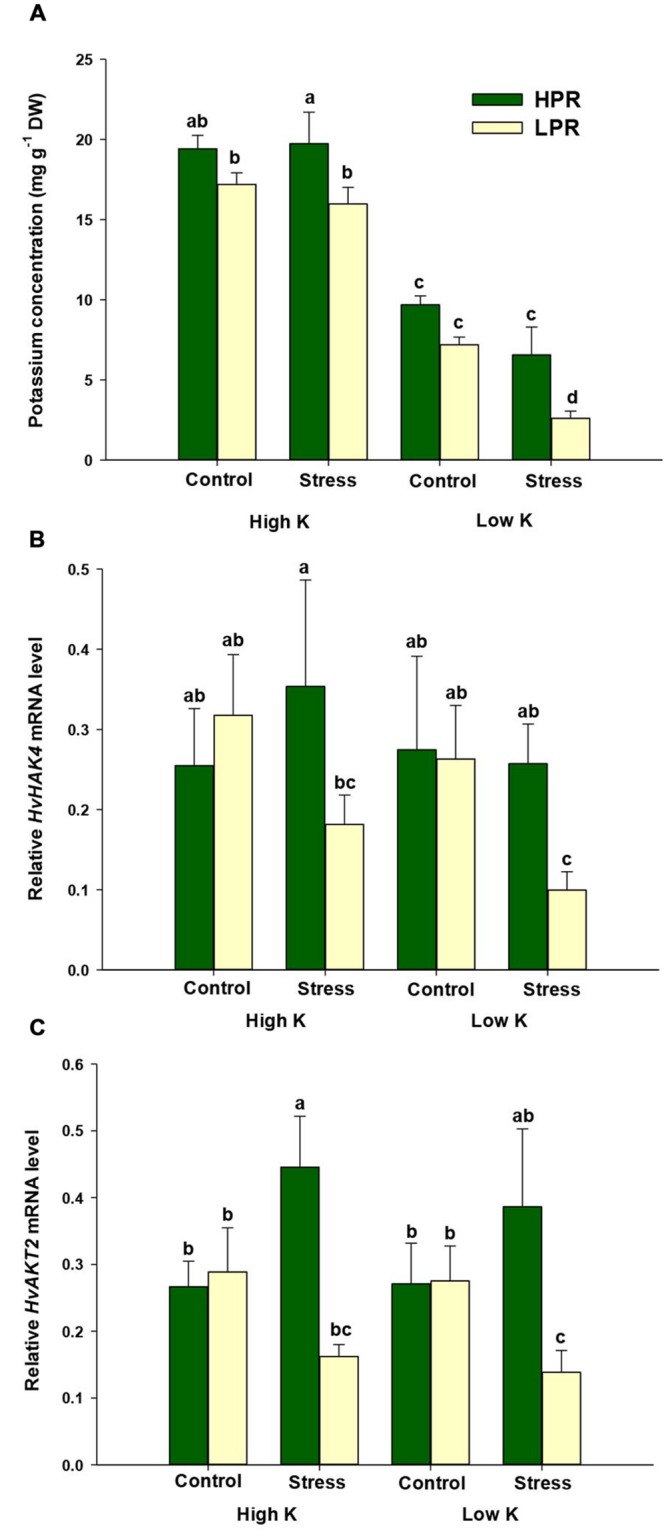
**Influence of K supply on K concentrations and gene expression of K transporters in flag leaves of barley during terminal drought stress. (A)** K concentrations, **(B)** relative *HvHAK4* mRNA levels, and **(C)** relative *HvAKT2* mRNA levels in flag leaves of the lines HPR and LPR. Plants were pre-cultured under sufficient water supply (control) or water limitation (stress) and under low or high K supply. Flag leaves from 12 weeks-old plants were harvested 12 days after imposing drought stress. Bars indicate mean ± SD. Different letters denote significant differences according to three-way ANOVA and Tukey’s test (*p* < 0.05; *n* = 6). *Ubi E2-17* (ubiquitin-conjugating enzyme) was used as reference gene.

Analysis of the K nutritional status indicated adequate K concentrations of 1.5–2.0% in flag leaves of barley plants supplemented with high K levels (**Figure 2A**). Under limiting K supply, K concentrations decreased to less than 1%, which is indicative of K deficiency in barley (Bergmann, 1992). When plants were subjected to drought stress, K concentrations in flag leaves of LPR decreased to <0.3% indicative of severe K deficiency. However, HPR did not show such a drop and finally maintained 2.5 times higher K concentrations in its flag leaves than LPR. In both lines the decrease in flag leaf K concentrations was accompanied by an increase in the concentrations of Mg and Ca (Supplementary Figure S1). The sharper drop of K levels in LPR was even reflected by a significant increase in Mg concentrations. This is in agreement with the typical response of K-deficient plants, which compensate for lower K uptake by enhanced uptake of other cations, in particular Ca and Mg (Marschner, 2012).

Since the high-affinity K transporter HvHAK4 and the K channel HvAKT2 have been proposed to mediate K^+^ uptake into growing leaf tissues, in particular in the leaf elongation zone in barley (Boscari et al., 2009), we determined expression levels of the corresponding genes. Neither of the two genes showed a consistent response to the K treatment (**Figures 2B,C**). However, imposing drought stress in LPR led to a decrease in mRNA levels of *HvHAK4* and *HvAKT2*, which was significant under low K supply. In HPR, transcript levels of *HvHAK4* remained rather constant, while those of *HvAKT2* even increased or tended to increase under low K supply. Thus, under limited K supply HPR retained not only more K than LPR, but probably maintained also its ability to incorporate K into the tissue of drought-stressed flag leaves, which was indicative for a superior physiological performance of HPR.

### Differential Response of Flag Leaf Primary Metabolism in HPR and LPR

Several studies have shown a strong impact of either K deficiency or drought stress on carbohydrate metabolism in leaves (Amtmann et al., 2008; Armengaud et al., 2009; Bowne et al., 2012; Silvente et al., 2012). Both, K deficiency and drought can lead to an accumulation of osmotically active carbohydrates in leaves (Cakmak et al., 1994; Wingler et al., 2006, 2009). By LC-MS we monitored metabolite levels and confirmed enhanced levels of glucose, fructose and mannitol in response to drought under both K treatments (**Table 1**). While this typical response to drought was evident in both lines, we focused on those metabolite changes that showed genotypical differences in particular under K-deficient growth conditions. For primarily chloroplast-localized metabolites, most dramatic genotypical differences were observed in concentrations of starch, NADPH, ATP, and to a weaker extent also for 3-PGA and PEP, as these metabolites and energy carriers responded more sensitively to drought and decreased to much lower levels in LPR than in HPR (**Table 1**). Comparing rather genotypical differences in metabolite changes under low K emphasized the higher sensitivity of LPR to drought, as in particular metabolites of the glycolysis pathway, such as glucose-6P, fructose-6P and 3-phosphoglycerate, were lower than in HPR (Supplementary Figure S2). Among TCA cycle metabolites only 2-oxoglutarate and fumarate showed a stronger decrease in LPR than in HPR under drought stress and concomitant low K. Proline, which typically increases under drought stress (Bowne et al., 2012) increased under drought only at low K supply in HPR, while it decreased under the same conditions in LPR, in which control levels of proline were unexpectedly high (**Table 1**). Among other drought-responsive amino acids Asn, Gln, and Phe (Bowne et al., 2012) increased not only under low K supply but also under drought stress, and this increase was more pronounced in LPR than in HPR. By contrast, Asp, Glu, and Gly showed no consistent changes according to stress treatments or genotype (**Table 1**). Thus, drought stress-induced changes in primary C/N metabolism were apparently more affected by K deficiency in LPR than in HPR, and in particular starch decreased under drought stress in a more pronounced way in LPR than in HPR.

**Table 1:** Influence of K supply on metabolite concentrations in flag leaves of barley during terminal drought stress.

		High K				Low K			
		Control		Stress		Control		Stress	
Pathways	Metabolite	HPR	LPR	HPR	LPR	HPR	LPR	HPR	LPR
Sugars	Glucose	3.0 c	3.3 c	12.2 b	19 a	3.4 c	5.4 c	19.4 a	23.2 a
	Fructose	6 b	7.4 b	22.9 a	23.7 a	6.4 b	8.7 b	22.9 a	30.7 a
	Sucrose	84.5 a	63.6 ab	49.2 ab	61 ab	55.1 ab	62.2 ab	38.8 b	35.8 b
	Starch	18.8 ab	16.9 bc	11.6 cd	4.4 ef	23.2 a	16.2 bcd	10.2 de	2.71 f
Sugar alcohol	Mannitol	33.6 cd	47.6 cd	85.38 bc	88.01 b	27.7 cd	11.7 d	153.9 a	94.68 b
Starch metabolism	Tre-6-P	0.8 cd	1.2 ab	1.2 bc	1.6 a	0.5 cd	0.7 cd	0.7 cd	0.4 d
	Sucrose-6-P	1.1 bc	1.3 abc	1.6 ab	1.9 a	1.2 bc	0.8 c	1.3 abc	0.8 c
	Hexose-6-P	73.5 ab	51.9 bc	73.8 ab	79.4 ab	80.7 a	25.2 cd	62.1 ab	21.2 d
	UDP-Glucose	39.1 a	40.9 a	32.1 ab	23.2 b	39.5 a	23.2 b	35.8 ab	19.8 b
	ADP-Glucose	0.2 a	0.06 bc	0.03 c	0.05 c	0.05 c	0.05 c	0.11 ab	0.04 bc
Calvin cycle	NADPH	1.2 bc	1.2 bc	1.4 bc	0.4 c	2.1 b	0.6 bc	3.5 a	0.7 bc
	ATP	19.2 a	14.5 abc	14.8 ab	1.8 d	8.6 bcd	3.9 cd	5.6 cd	2.1 d
Glycolysis	3PGA	515 a	417 ab	325 bc	46.8 e	214 cd	33.6 e	144 de	24.4 e
	PEP	19.2 ab	27.4 a	5.7 bc	0.2 c	19.2 ab	1.1 c	4.9 bc	0.8 c
TCA cycle	Oxoglutarate	301 abc	365 a	255 abc	167.4 cd	335 ab	224 bc	193 c	48.6 d
	Fumarate	4070 a	3189 bcd	3279 bc	2592 cd	3338 b	2510 de	3112 bcd	1704 e
	Citrate	13720 a	10108 b	6687 bc	5388 bc	3990 bc	3319 c	3892 bc	4710 bc
	Isocitrate	10055 a	11088 a	10685 a	9561 a	3440 b	4788 b	3379 b	3126 b
	Malate	12979 a	9743 b	6420 bc	6204 bc	5324 bc	2312 bc	4433 bc	4805 bc
Amino acids	Asn	36.3 cd	51.2 bc	23.4 d	68.9 a	51.2 bc	64.6 ab	54.2 ab	70.63 a
	Gln	58.2 b	84.1 b	74.4 b	128 b	131 b	132 b	64.7 b	380.76 a
	Gly	165 a	128 abc	90.7 c	159 ab	128 abc	119 bc	95 c	96.83 c
	Asp	915 a	1014 a	627 b	434 bcd	518 bc	457 bcd	363 cd	221 d
	Glu	6789 a	3009 b	3939 b	4893 ab	3770 b	3625 b	3698 b	3827 b
	Pro	13.2 c	14.7 c	12.5 c	13.8 c	18.9 c	59.3 a	33 b	16.17 c
	Phe	34.8 d	29.9 d	28.3 d	42.7 d	59.8 c	76.2 b	37.9 d	122.4 a

*Concentrations of soluble and insoluble sugars, sugar alcohols, amino acids, and primary metabolites were measured in flag leaves of the lines HPR and LPR. Plants were pre-cultured under sufficient water supply (control) or water limitation (stress) and under low or high K supply. Flag leaves from 12 weeks-old plants were harvested 12 days after imposing drought stress. Bars indicate mean ± SD. Different letters denote significant differences according to 3-way ANOVA and Tukey’s test (*p* < 0.05; *n* = 4–6). Tre-6-P, trehalose-6-phosphate; Sucrose-6-P, sucrose-6-phosphate; Hexose-6-P, hexose-6-phosphate; ADP-Glc, ADP-glucose; 3PGA, 3 phosphpoglycerate; PEP, phosphoenolpyruvate; UDP-Glc, UDP-glucose; NADPH, nicotinamide adenine dinucleotide phosphate; ATP, adenosine triphosphate; Asn, asparagine; Gln, glutamine; Gly, glycine; Asp, aspartate; Glu, glutamate; GABA, gamma aminobutyric acid; Pro, proline; Phe, phenylalanine.*

**FIGURE 3:**
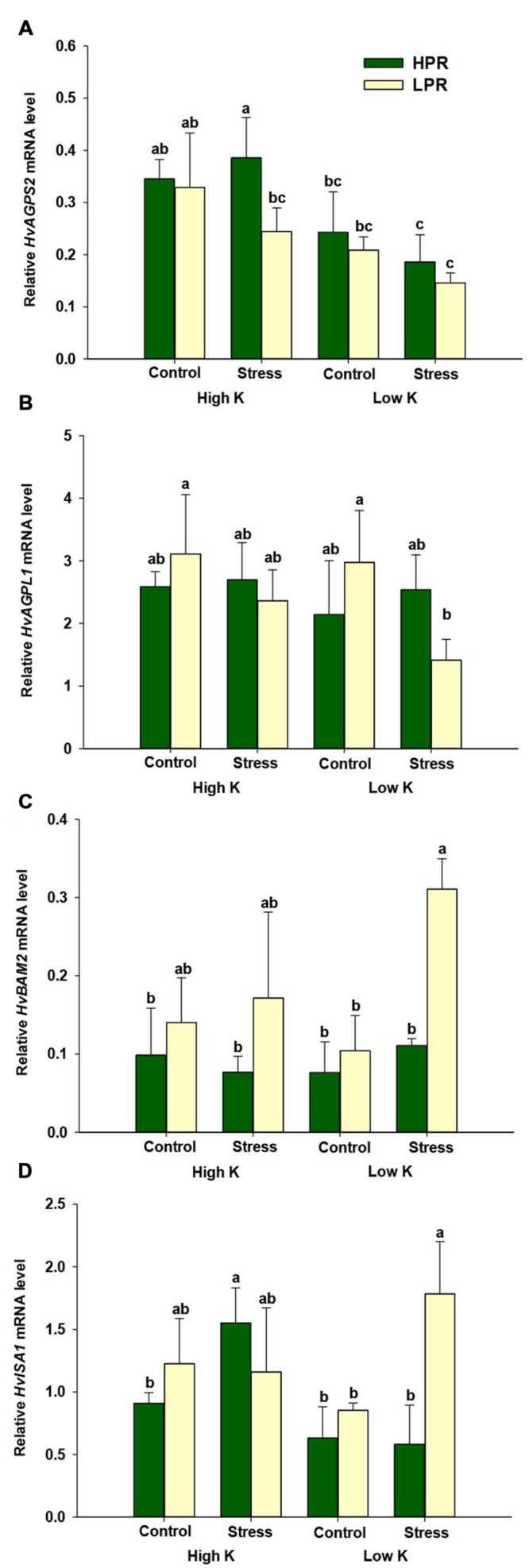
**Influence of K supply on the expression of genes involved in starch biosynthesis or degradation in flag leaves of barley under drought stress.** Relative mRNA levels of **(A)** ADP-glucose pyrophosphorylase small subunit (*HvAGPS2*), **(B)** ADP-glucose pyrophosphorylase large subunit (*HvAGPL1*), **(C)** β-amylase (*HvBAM2*), and **(D)** isoamylase (*HvISA1*) in flag leaves of the lines HPR and LPR. Plants were pre-cultured under sufficient water supply (control) or water limitation (stress) and under low or high K supply. Flag leaves from 12 weeks-old plants were harvested 12 days after imposing drought stress. Bars indicate mean ± SD. Different letters denote significant differences according to three-way ANOVA and Tukey’s test (*p* < 0.05; *n* = 4–6). *Ubi E2-17* (ubiquitin-conjugating enzyme) was used as reference gene.

To learn more about the processes leading to genotypical differences in starch in the two lines, we investigated the expression levels of two genes in starch biosynthesis, ADP-glucose pyrophosphorylase small subunit (*HvAGPS2*) and ADP-glucose pyrophosphorylase large subunit (*HvAGPL1*). However, besides a weak trend of *HvAGPS2* mRNA levels to decrease under low K and drought stress, there were no consistent differences between the two lines (**Figures 3A,B**). We then monitored beta-amylase (*HvBAM2*) and isomerase (*HvISA1*), which encode key components in the starch degradation pathway (Radchuk et al., 2009). In fact, transcript levels of both genes were strongly up-regulated in LPR only under concomitant drought and K deficiency, while otherwise differences between the two lines were small or insignificant under control conditions (**Figures 3C,D**). This observation suggested that LPR induces a higher turnover of starch than HPR under concomitant K deficiency and drought stress.

### Role of K in ABA Homeostasis During Drought Stress

**FIGURE 4:**
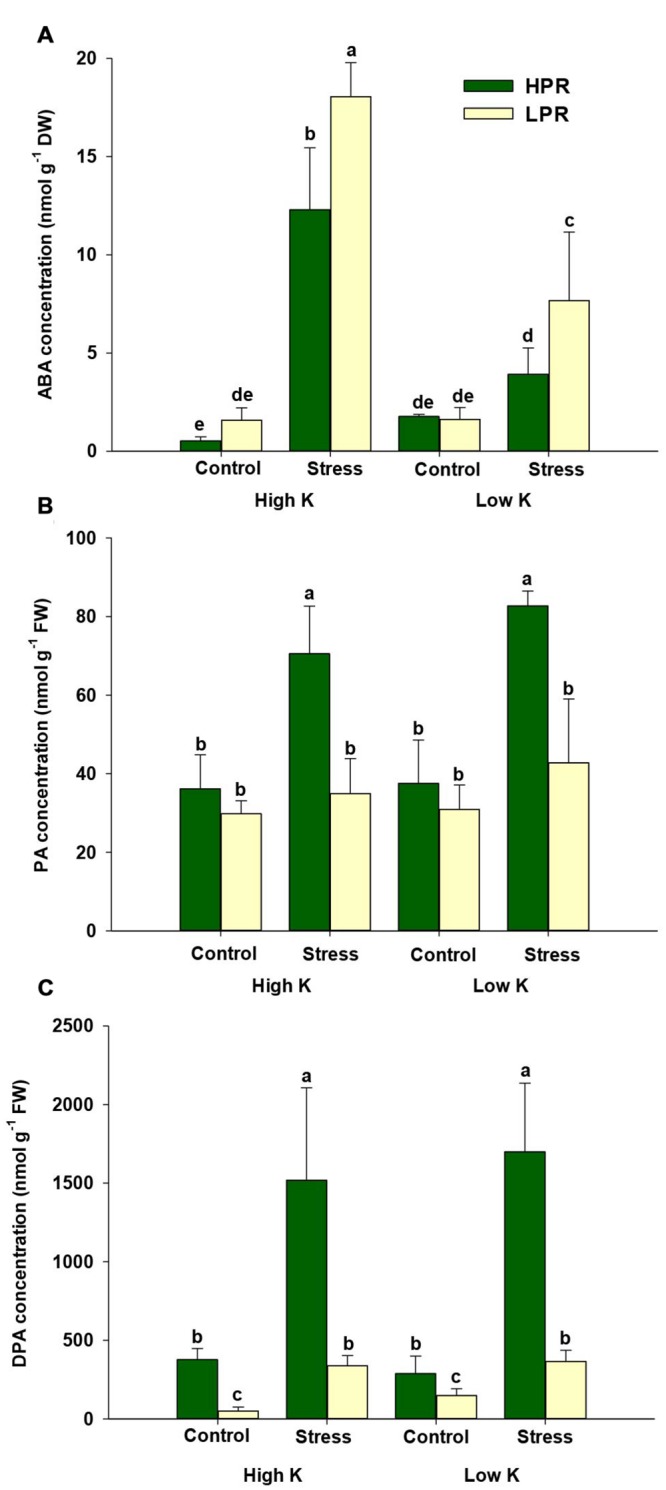
**Influence of K supply on ABA homeostasis in flag leaves of barley during terminal drought stress.** Concentrations of **(A)** abscisic acid (ABA), **(B)** phaseic acid (PA), and **(C)** dehydro-phaseic acid (DPA) in flag leaves of the lines HPR and LPR. Plants were pre-cultured under sufficient water supply (control) or water limitation (stress) and under low or high K supply. Flag leaves from 12 weeks-old plants were harvested 12 days after imposing drought stress. Bars indicate mean ± SD. Different letters denote significant differences according to three-way ANOVA and Tukey’s test (*p* < 0.05; *n* = 6).

Based on the higher starch levels in K- and drought-stressed HPR leaves, we further investigated ABA-related responses in both lines, since a cross talk has been postulated between starch and the ABA signaling pathway (Fujii et al., 2009; Seiler et al., 2011). For this purpose, we quantified ABA and its degradation products by UPLC-MS/MS as well as transcript levels of two key genes from the ABA biosynthesis and degradation pathways. In both lines, the ABA concentration strongly increased under drought at adequate K supply (**Figure 4A**). Unexpectedly, this drought-induced increase in ABA was compromised rather than promoted under concomitant K deficiency. At both K levels, the line HPR accumulated significantly less ABA under drought in comparison to the line LPR. In contrast, HPR accumulated higher levels of the degradation products phaseic acid (PA) and in particular of dehydro-phaseic acid (DPA) than LPR under drought stress at either K supply (**Figures 4B,C**). This suggested that the lower ABA concentration in HPR was at least in part related with a higher turnover of ABA.

Based on the importance of cytokinins in abiotic stress responses (Zwack and Rashotte, 2015), we also measured different forms of cytokinins but were able to quantify only isopentenyladenine and its ribosylated form in flag leaves. While isopentenyladenine-riboside, which represents a transport form, showed less consistent differences between the two lines, isopentenyladenine concentrations decreased under drought stress in both lines. In LPR this decrease was more pronounced in particular when K supply was low (Supplementary Figure S3). Thus, isopentenyladenine concentrations behaved almost oppositely to ABA (**Figure 4A**), supporting the view of an antagonistic interaction between these two hormone classes under abiotic stresses (Nishiyama et al., 2011).

**FIGURE 5:**
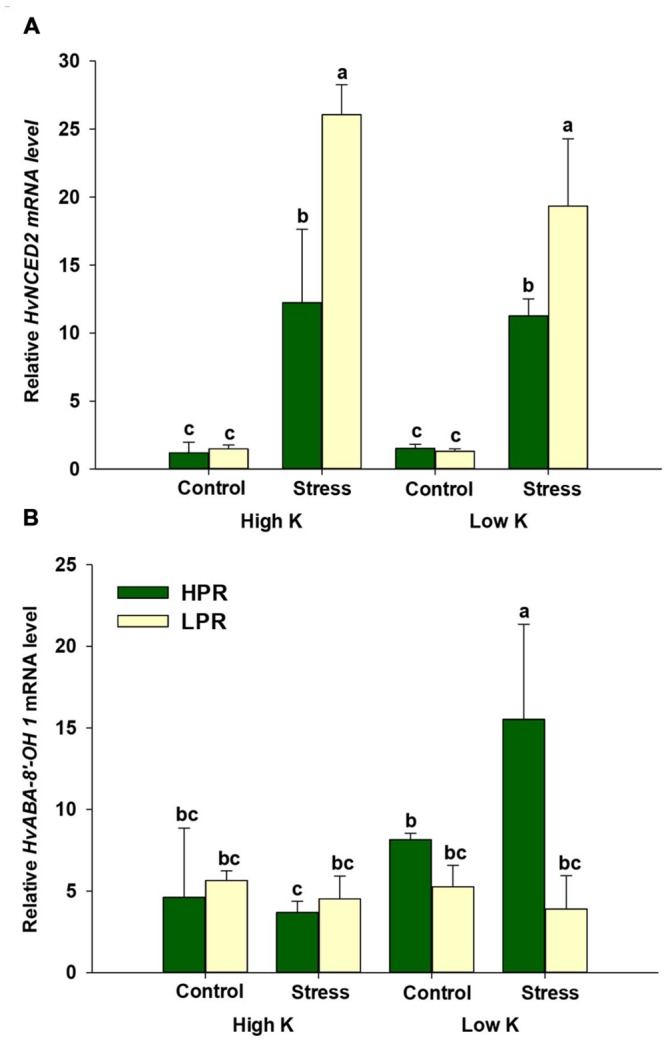
**Influence of K supply on the expression of genes involved in ABA biosynthesis and degradation in flag leaves of barley under drought stress.** Relative mRNA levels of **(A)** 9-*cis*- epoxy-carotenoid dioxygenase (*HvNCED2*) and **(B)** ABA-8′-hydroxylase 1 (*HvABA-8*′*-OH1*) in flag leaves of the lines HPR and LPR. Plants were pre-cultured under sufficient water supply (control) or water limitation (stress) and under low or high K supply. Flag leaves from 12 weeks-old plants were harvested 12 days after imposing drought stress. Bars indicate mean ± SD. Different letters denote significant differences according to three-way ANOVA and Tukey’s test (*p* < 0.05; *n* = 4–6). *Ubi E2-17* (ubiquitin-conjugating enzyme) was used as reference gene.

In the ABA biosynthesis pathway, the gene product of *9-cis-EPOXYCAROTENOID DIOXYGENASE 2* (*HvNCED2*) catalyzes the conversion of 9-*cis*-violaxanthin to xanthoxin, an important precursor in ABA biosynthesis. Transcript levels of this gene revealed a higher abundance when plants were subjected to drought stress. In LPR, however, this increase was significantly higher than in HPR (**Figure 5A**), which was in agreement with the higher ABA levels in LPR (**Figure 4A**). On the other hand, monitoring *ABA-8*′*-HYDROXYLASE 1* (*HvABA-8*′*-OH1*), mediating the key step in ABA degradation, showed increased transcript levels in HPR under drought stress and concomitant K deficiency (**Figure 5B**). Thus, comparing the accumulation of ABA and its degradation products with gene expression levels suggested that ABA accumulation in LPR was more related to ABA biosynthesis, while in HPR ABA levels were subject to accelerated degradation, at least under K-deficient conditions.

## Discussion

In mineral plant nutrition, there is an outstanding role of K in the regulation of the water status and drought stress responses, however, this knowledge is largely based on experiments in which K was supplemented to drought-stressed plants (Cheong et al., 2007; Damon and Rengel, 2007; Römheld and Kirkby, 2010; Wang et al., 2013). Evidence for a genetically controlled component for the beneficial role of K in drought stress tolerance is still lacking. Based on the comparison of two barley lines that differ in drought-induced leaf senescence, we tackled the question whether and to what extent genotypic differences in drought tolerance are related to the K nutritional status in leaves. Comparing the drought stress responses of these two genotypes at different K supply levels indicated that the ability of the line HPR to retain more K in flag leaves is associated with lower ABA and higher starch concentrations. Thus, superior tolerance to drought may benefit from higher K retention in flag leaves and hence a larger reserve of carbohydrates required for osmotic adjustment. Our study further indicates that the relation between K and drought-induced ABA accumulation and its turnover is of physiological relevance particularly in drought-stressed cereal crops.

### A Protective Role of K in Drought Stressed-Induced Leaf Senescence

To investigate the role of K in terminal drought stress, the two lines HPR and LPR were subjected to drought under high and low K supply and found to differ in the water status of their flag leaves. The lower K levels in LPR resulted in greater water loss and accelerated chlorophyll degradation (**Figure 1**). These results are in agreement with findings reported in sunflower, in which K starvation decreased leaf water potential or enhanced sensitivity to drought stress and impaired photosynthesis (Lindhauer, 2007; Benlloch-González, 2010). This drought-dependent physiological trait was confirmed by a molecular marker for leaf senescence (Krupinska et al., 2002; Shi et al., 2012), as *HvS40* transcript levels were strongly upregulated under K limitation and drought stress only in LPR but not in HPR (**Figure 1C**). These measures clearly showed that flag leaves of the line HPR experienced less severe drought stress despite low K supply. Nevertheless, chlorophyll degradation in HPR plants tended to be lower under low K compared to high K. Presumably, HPR plants have been “primed” to stress due to the low K treatment, to which plants were exposed from the beginning of the culture. This would explain why in HPR *HvS40* expression was already induced under low K control and why the low K stress treatment did not lead to further increases in *HvS40* expression and chlorophyll degradation. As revealed by mineral element analysis, low K supply decreased K concentrations in flag leaves far below critical deficiency levels which may vary between 20 and 50 mg g^-1^ in non-stressed cereal leaves (**Figure 2A**; Bergmann, 1992; Marschner, 2012). Notably, only flag leaves of LPR dropped even below 3 mg K g^-1^ and thus to a 2.5 times lower level than those in HPR. This superior K nutritional status of HPR was further supported by monitoring transcript levels of two K transporters, which decreased under drought stress in LPR but not in HPR (**Figures 2B,C**). This held true not only for K-deficient but also for K-sufficient plants, which may indicate that these two genes responded even more sensitively to drought than to K in a genotype-dependent manner. As K channels and transporters play a crucial role in stabilizing the membrane potential in leaf cells, especially under drought stress, K may not only have acted as an osmoticum but also promoted water transport via aquaporins, which are often co-regulated with K channels as part of the cellular osmoregulation (Liu et al., 2006; Wang et al., 2013). Based on the close association between the K nutritional status, the relative water content and drought-induced leaf senescence, we postulated that in flag leaves K takes over a protective role against drought stress and that the higher K retention in HPR in flag leaves may be a determinant of drought stress tolerance.

In previous studies a tight link has been reported between drought tolerance and carbohydrate metabolism in cereals (Kameli and Loseldelta, 1993; Seki et al., 2007; Mohammadkhani and Heidari, 2008). In particular the drought stress-induced increase in primary metabolites acting as compatible solutes, such as mannitol or sugars, has been associated with a decrease in starch (Barnabás et al., 2008; Naser et al., 2010), emphasizing the relevance of starch in safeguarding the carbohydrate reserve for the synthesis of compatible solutes. With regard to the prominent role of K in primary carbon and especially starch metabolism (Hermans et al., 2006; Marschner, 2012), we first focused on those metabolites which were significantly different between HPR and LPR under drought stress at low K supply (**Table 1**; Supplementary Figure S1). Among those were not only carbon metabolites but also amino acids, especially Asn, Gln and Phe, that were previously shown to be highly responsive to drought stress in wheat leaves (Bowne et al., 2012). In addition appeared a prominent number of metabolites related to carbon assimilation and energy transfer in the chloroplast, including the energy carriers NADPH and ATP, which mostly remained along with starch at higher levels in HPR than in LPR (Supplementary Figure S1). These results suggested that ATP synthesis and NADPH formation are critical for maintaining functional metabolism under stress. Moreover, also key metabolites of the TCA, i.e., 2-oxoglutarate and fumarate, were less severely affected by drought and K deficiency in HPR. By monitoring metabolite levels and enzyme activities in K-starved *Arabidopsis* leaves, Armengaud et al. (2009) identified pyruvate kinase as a highly K-sensitive step in glycolysis. Although we could not measure pyruvate here, elevated levels of other glycolysis intermediates, namely glucose-6P, fructose-6P and 3-PGA, were much less affected by drought and K deficiency in HPR than in LPR (**Table 1**; Supplementary Figure S1). These changes suggested that HPR profited from the larger starch reserve in the chloroplast to continue fueling carbohydrate metabolism.

K is involved in the activation of many enzymes, and in particular the activity of starch synthase is highly dependent on univalent cations, among which K^+^ is most effective (Marschner, 2012). In this regard, transcriptome profiling of two Tibetan wild barley genotypes subjected to low K identified a marked alteration of several enzymes, which are involved in starch and sucrose metabolism (Zeng et al., 2014). Taking here a closer look at the transcript level of genes involved in starch biosynthesis and degradation showed that genes of two enzymes involved in starch biosynthesis, *HvAGPS2* and *HvAGPL1*, showed little or no difference between the two lines (**Figures 3A,B**). In contrast, mRNA levels of genes involved in starch degradation, i.e., *HvBAM2* and *HvISA1* (Smith et al., 2005), showed a strong up-regulation under drought and K deficiency only in LPR (**Figures 3C,D**). Thus, differences in starch levels in the two genotypes most likely resulted from enhanced starch degradation in LPR finally causing lower starch concentrations (**Table 1**). Taken together, we concluded that the higher retention of K in drought-stressed flag leaves of HPR prevented an accelerated degradation of starch, favored elevated carbon pools and thereby contributed to superior drought stress tolerance.

### Drought-Dependent ABA Metabolism is Subject to Genotypic Differences

Terminal drought is characterized by an elevated production of the phytohormone ABA which plays an important role not only for developmental senescence but also for stress-induced senescence (Distelfeld et al., 2014). Consistent with the observation that ABA concentrations in leaves increase under drought stress (Ikegami et al., 2009) and barley lines maintaining optimum ABA homeostasis show better water use efficiency (Seiler et al., 2014), ABA levels in flag leaves increased under drought to a larger extent in LPR than in HPR (**Figure 4A**), which coincided with an accelerated induction of the senescence marker gene *HvS40* in LPR (**Figure 1C**). Unexpectedly, this increase under drought was even higher in K-sufficient than in K-deficient plants which may reflect in part a higher responsiveness of K-sufficient plants to the drought treatment. To verify whether ABA levels were primarily a result of enhanced ABA biosynthesis, transcript levels of *HvNCED2* were determined and found to strongly respond to drought stress irrespective of the K treatment (**Figure 5A**). This drought stress-induced increase in mRNA levels was significantly lower in HPR than in LPR and closely reflected the differences in ABA concentrations between the two lines (**Figure 4A**). It was thus concluded that the genotypic differences in ABA levels in response to drought were primarily due to the transcriptional regulation of *HvNCED2*.

Abscisic acid homeostasis in plant tissues results from a balance between *de novo* synthesis, export, conjugation, and degradation (Cutler and Krochko, 1999; Boursiac et al., 2013). While the contribution of export and conjugation to ABA homeostasis is still hard to quantify, ABA degradation has proven substantial. In the majority of plant tissues, catabolic inactivation of ABA proceeds via 8′-hydroxy-ABA, which is spontaneously converted to PA. Then, PA is further reduced to DPA (Cutler and Krochko, 1999). In this study, the concentrations of both degradation products strongly increased in drought-stressed HPR plants regardless of the amount of K added to the substrate (**Figures 4B,C**). This suggested that the lower ABA levels in drought-stressed HPR relative to LPR plants may have been due to an accelerated degradation of ABA in HPR. Therefore, mRNA levels of *HvABA8*′*-OH* were determined, which takes in a central role in ABA catabolism (Sreenivasulu et al., 2012; Cai et al., 2015). Notably, the mRNA level of *HvABA8*′*-OH1* in the line HPR was up-regulated under water scarcity only when K supply was low (**Figure 5B**). Presuming that these higher transcript levels also resulted in a higher activity of *HvABA8*′*-OH1*, the activation of the corresponding catabolic enzyme may have contributed to the observed decrease of ABA in K-deficient versus K-sufficient HPR plants under drought stress (**Figure 4A**). A similar observation has been made by Seiler et al. (2011) in flag leaves of senescing barley, where PA and DPA accumulated to several-fold higher levels than ABA, which also coincided with elevated mRNA levels of *HvABA8*′*-OH1* under terminal drought stress. Such a regulatory link may be triggered by regulatory proteins like the NUCLEAR PROTEIN X1 (NPX1), which is induced by K deficiency in *Arabidopsis* and modulates genes involved in ABA homeostasis (Kim et al., 2009). However, the paralog to *AtNPX1* in barley could not yet be identified. Alternatively, lower ABA levels and accelerated ABA degradation may also have been related to the higher levels of the cytokinin isopentenyladenosine (Supplementary Figure S3), since higher endogenous levels of cytokinins have been shown to decrease the sensitivity of plants to ABA (Nishiyama et al., 2011). Taken together, the K treatment and the resulting nutritional status had a minor impact on drought-induced ABA metabolism in flag leaves. However, genotypic differences between HPR and LPR in ABA homeostasis were significant and the higher turnover of ABA in K-deficient and drought-stressed HPR may have contributed to the superior drought tolerance observed in this line.

## Conclusion

The present study provides correlative evidence that the genotype HPR performed better under drought stress due to its superior K nutritional status and a more balanced ABA homeostasis and carbohydrate metabolism. So far, such relations had to be deduced from independent studies dealing either with K deficiency or with drought stress. Later senescence in the more drought-tolerant line HPR likely profited from a higher retention of K in flag leaves safeguarding starch for the sake of a better metabolic adjustment to drought. Genotypic differences were also reflected by a higher turnover of ABA in the tolerant line HPR, in which a superior K nutritional status was associated with ABA hydrolysis. Thus, a high K nutritional status in flag leaves likely contributes to a higher tolerance against drought-induced leaf senescence by promoting ABA degradation but attenuating starch degradation. In breeding approaches for drought-tolerant barley, genotypic differences in flag leaf K concentrations may even serve as markers for the development of drought-tolerant lines.

## Author Contributions

SH conducted the experiments and analyzed data. MH supervised the metabolite analysis. CS analyzed gene expression data. NS and NW designed the experiments and evaluated the data. SH, NS, and NW wrote the article.

## Conflict of Interest Statement

The authors declare that the research was conducted in the absence of any commercial or financial relationships that could be construed as a potential conflict of interest.
