# Supplementary material for: A Potential Role of Flag Leaf Potassium in Conferring Tolerance to Drought-Induced Leaf Senescence in Barley
Source: Front Plant Sci. 2016 Feb 26;7:206. doi: 10.3389/fpls.2016.00206 (PMC4768371; doi:10.3389/fpls.2016.00206)
Supplement: Supplementary file 1 [file Data_Sheet_1.PDF]

Supplementary Table S1. List of primers used for qRT-PCR.

Gene	Forward primer	Reverse primer	Reference
HvS40	ATGTACCAACGACGCGAAG	ATGAATCCGGTCATCCTGAG	
HvHAK4	AAGAAGTTTGCGGTCGATGTTGGC	AGCAATAGGGCTTCACAGGTAGCA	Boscari et al, 2009
HvAKT2	ACCCAAGAGCACATGAGGATGCTA	ACATGCCACGGAGATGACTGGATT	Boscari et al, 2009
HvAGP-L1	AAAGTGGCAGCGAGCAAACAAA	GACAGAGGCAGCGGGAAAACC	Seiler et al, 2011
HvAGP-S2	TCCCATGCAGCAAGTTCACCAA	TTCTTTCCGCCGACGACACTTC	Seiler et al, 2011
HvBAM2	GCGCTCCTTGCCCCTGTGG	CTTGGCGGCTTATTTCCTGTGC	Seiler et al, 2011
HvISA1	CACAAAAGGGGGCAACAACAAT	GGCCAAGACCCTCGCACTCC	Seiler et al, 2011
HvNCED2	CATGGAAAGAGGAAGTTG	GAAGCAAGTGTGAGCTAAC	Millar et al., 2006
HvABA8'OH	AGCACGGACCGTCAAAGTC	TGAGAATGCCTACGTAGTG	Millar et al., 2006
HvIPT1	TCGGTGTAGATGGAACACCA	TTGCGGCAATATGAAGTCAG	

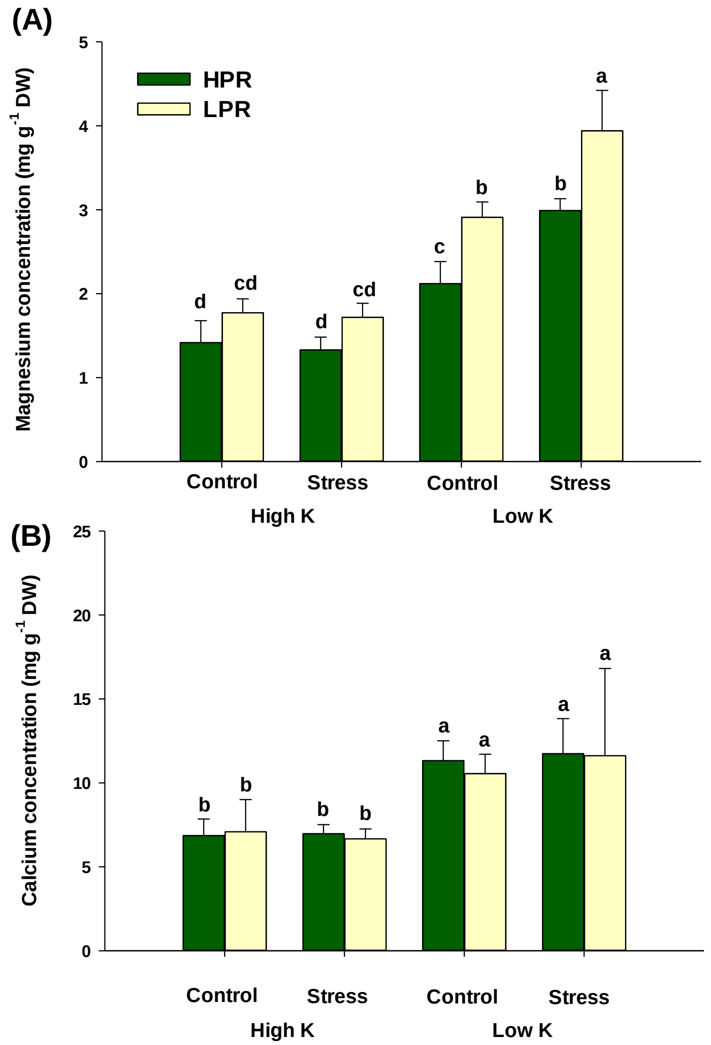

Supplementary Figure S1. Influence of K supply on Mg and Ca concentrations in flag leaves of barley during terminal drought stress. Concentrations of A) magnesium and B) calcium in flag leaves of the lines HPR and LPR. Plants were pre-cultured under sufficient water supply (control) or water limitation (stress) and under low or high K supply. Flag leaves from 12 weeks-old plants were harvested 12 days after imposing drought stress. Bars indicate means ± SD. Different letters denote significant differences according to 3-way ANOVA and Tukey's test ($p<0.05$; $n=6$).

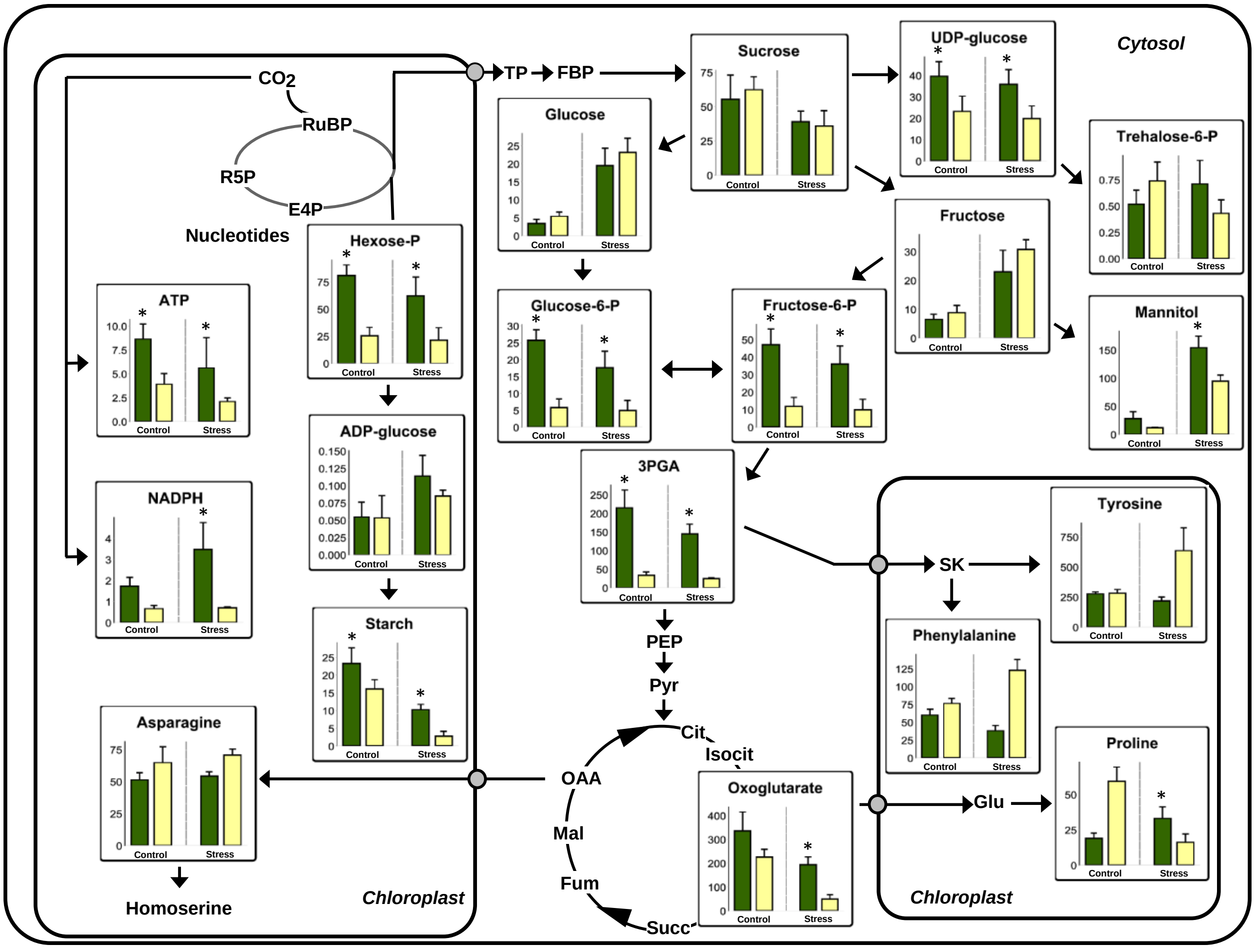

Supplementary Figure S2. Metabolite concentrations in flag leaves of the two barley lines HPR (green bars) and LPR (yellow bars) under low K supply. Simplified scheme of metabolic pathway showing the levels of the primary metabolites determined by GC-MS in plants grown under either sufficient water supply (control) or water limitation (stress). Bars indicate means ± SD. Different letters denote significant differences according to 3-way ANOVA and Tukey's test ($p<0.05$; n=4-6). Abbreviations are as follows: OAA: oxaloacetate, R5P: ribulose-5-phosphate, TP, triose-phosphate, RuBP: ribulose-1,5-bisphosphate, E4P: erythrose-4-phosphate, ATP: adenosine triphosphate, NADPH: nicotinamide adenine dinucleotide phosphate, 3PGA: 3-phosphoglycerate, Pyr: Pyruvate, PEP: phosphoenolpyruvate, Succ: succinate, Glu: glutamate, Cit: citrate, Isocit: isocitrate, Glu: glutamate, SK: shikimate pathway. Concentrations of metabolites were calculated based on nmol/g FW. Asterisks indicate significant differences between HPR and LPR according to 3-way ANOVA and Tukey's test ($p<0.05$; n=6).

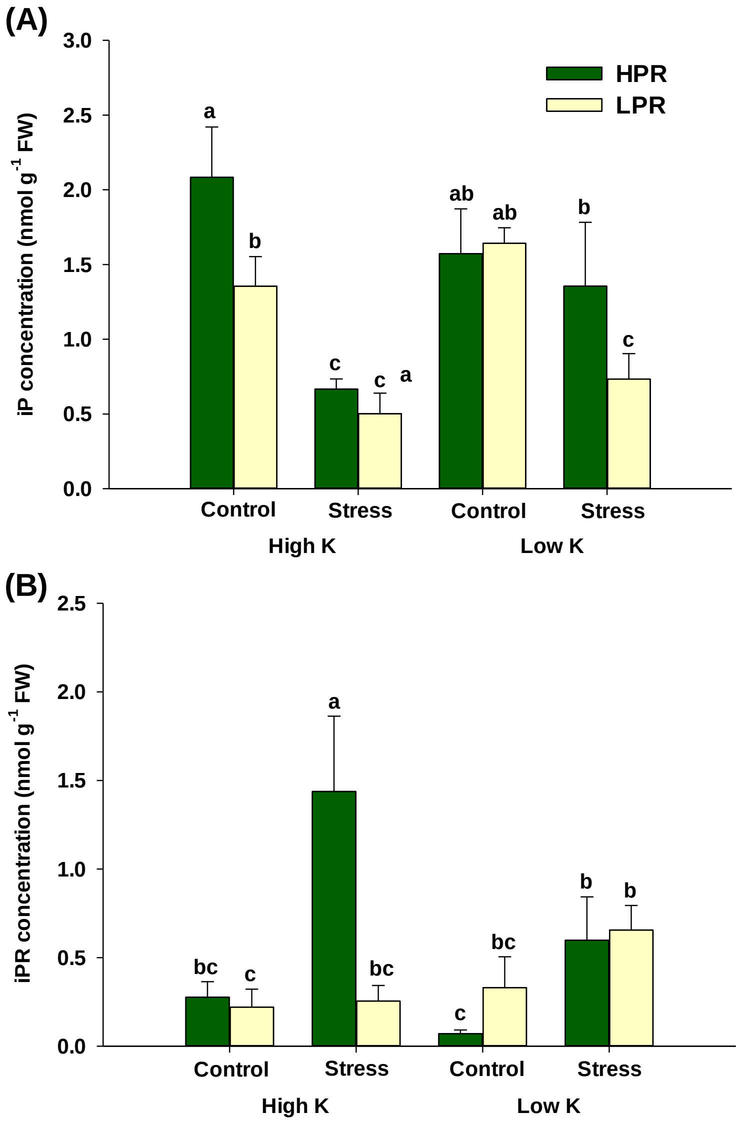

Supplementary Figure S3. Influence of K supply on cytokinin concentrations in flag leaves of barley during terminal drought stress. Concentrations of A) isopentenyladenine (iP), B) isopentenyladenine riboside (iPR) in flag leaves of the lines HPR and LPR. Plants were pre-cultured under sufficient water supply (control) or water limitation (stress) and under low or high K supply. Flag leaves from 12 weeks-old plants were harvested 12 days after imposing drought stress. Bars indicate means ± SD. Different letters denote significant differences according to 3-way ANOVA and Tukey's test ($p<0.05$; $n=6$).
